# Impact of preoperative biliary drainage on postoperative outcomes in patients who undergo major hepatectomy after portal vein embolization for perihilar cholangiocarcinoma

**DOI:** 10.1007/s00595-025-03080-4

**Published:** 2025-07-08

**Authors:** Noriyuki Kitagawa, Akira Shimizu, Koji Kubota, Tsuyoshi Notake, Takahiro Yoshizawa, Kiyotaka Hosoda, Hikaru Hayashi, Shigeki Hayashi, Yuji Soejima

**Affiliations:** Division of Gastroenterological, Hepato-Biliary-Pancreatic, Transplantation and Pediatric Surgery, Department of Surgery, Shinshu University School of Medicine, 3-1-1 Asahi, Matsumoto, Nagano 390-8621 Japan

**Keywords:** Preoperative biliary drainage, Obstructive jaundice, Perihilar cholangiocarcinoma, Major hepatectomy, Portal vein embolization

## Abstract

**Purpose:**

The influence of preoperative biliary drainage (PBD) and portal vein embolization (PVE) on the occurrence of post-hepatectomy liver failure (PHLF) remains unclear. We evaluated their influence on postoperative outcomes, focusing on PHLF, in patients who underwent major hepatectomy for perihilar cholangiocarcinoma (PHCC).

**Methods:**

A total of 240 patients underwent major hepatectomy for PHCC between January 1990 and March 2021. We evaluated the influence of PBD on short-term outcomes in all patients and in a subgroup (*n* = 111) that received PVE.

**Results:**

Although the incidence of grade B/C PHLF in patients with PBD was higher than that in those without PBD, a multivariable analysis identified PVE (OR 3.98, 95% CI 1.9–8.4; *p* < 0.001) and organ/space surgical site infection (SSI) (OR 3.48, 95% CI 1.6–7.4; *p* = 0.001), but not PBD, as independent risk factors for grade B/C PHLF. A multivariate analysis of patients who underwent PVE revealed that organ/space SSI was an independent risk factor for grade B/C PHLF (OR 4.5, 95% CI 1.6–12.7; *p* = 0.005).

**Conclusion:**

PBD did not have a negative impact on the occurrence of PHLF in patients undergoing PVE for an initially inadequate future liver remnant volume, provided that appropriate antimicrobial prophylaxis was selected.

**Supplementary Information:**

The online version contains supplementary material available at 10.1007/s00595-025-03080-4.

## Introduction

Despite recent advances in pharmacotherapy and radiotherapy, surgical resection remains the only curative treatment for biliary tract malignancies [1–3]. Although major hepatectomy combined with resection of the extrahepatic bile duct is commonly performed for perihilar cholangiocarcinoma (PHCC), this procedure is technically demanding and has high mortality and morbidity rates [2–5]. Septic complications after hepatectomy are a major problem, and failure to control the focus of sepsis may cause hepatic failure or death.

Preoperative biliary drainage (PBD) is considered necessary for most patients with biliary tract malignancies requiring hepatectomy, and is associated with higher mortality rates in jaundiced patients than in those with normal livers [6]. However, PBD increases the incidence of postoperative infectious complications and does not reduce mortality or morbidity after liver resection [7–9]. In contrast, one study did not find a significant association between PBD and the development of postoperative infectious complications [10], whereas another reported a conflicting risk assessment of PBD for morbidity and mortality between right- and left-sided hepatectomy in patients with PHCC [11]. Therefore, the debate regarding the benefits of biliary drainage before hepatobiliary resection, particularly regarding the intended surgical procedure, remains unresolved.

Portal vein embolization (PVE) is the current reference standard for increasing the future liver remnant (FLR) to prevent post-hepatectomy liver failure (PHLF), the most serious and life-threatening complication after major hepatectomy [12]. These procedures are administered to patients with a relatively small FLR who are candidates for major hepatectomy such as right hemihepatectomy and right- or left-trisectionectomy. However, the relationship between PBD and PVE and their influence on PHLF are insufficiently understood.

We aimed to evaluate the influence of PBD on postoperative short-term outcomes in this difficult clinical setting, including in patients who experienced PHLF associated with major hepatectomy for PHCC, with special attention paid to those who received PVE.

## Methods

We enrolled 240 consecutive patients who underwent major hepatectomy for PHCC at the Department of Surgery, Shinshu University Hospital between January 1990 and March 2021. Detailed patient information was entered into a database that included clinical characteristics, preoperative nutritional status, procedures for biliary drainage, duration of biliary drainage, results of preoperative surveillance biliary cultures, surgical outcomes, results of postoperative surveillance cultures of abdominal drainage fluid, and postoperative short-term outcomes, such as morbidity and mortality. This study was approved by the Ethics Committee of Shinshu University (registration number: 4598) and was conducted in accordance with the Declaration of Helsinki (2013 update).

### Preoperative bile cultures and perioperative antibiotic treatment

For patients with clinical obstructive jaundice, percutaneous transhepatic biliary drainage (PTBD), endoscopic nasobiliary drainage (ENBD), endoscopic biliary stenting (EBS), or a combination of these were preferentially administered considering the FLR [13, 14]. External bile drained from the PTBD or ENBD catheter was sampled twice weekly for aerobic and anaerobic bacteria during the preoperative period. Antimicrobial prophylaxis (AMP) was determined based on the results of the final preoperative surveillance bile cultures as follows: if positive, AMP was selected according to the antibiotic susceptibility of the identified bacterial species [15]. If samples tested positive for two or more bacteria, one or two antibiotics were selected to ensure susceptibility across all bacteria. If cultures were negative, cephamycin was administered. The patients who did not undergo PBD were treated in the same manner as those with negative preoperative bile culture results.

AMP was administered intravenously 30 min before the initial skin incision and was repeated every 4 h during surgery. Additional antibiotics were administered for 3 days after surgery. When methicillin-resistant *Staphylococcus aureus* (MRSA) or *Enterococcus* species susceptible only to vancomycin were identified, vancomycin and other antibiotics against Gram-negative rods were selected for prophylaxis.

### Indications for and selection of hepatectomy

The type of hepatectomy was selected based on the results of preoperative imaging studies, including cholangiography, multidetector-row computed tomography (MDCT), magnetic resonance imaging (MRI), and intraductal ultrasonography. At our institute, right or left hemihepatectomy is generally performed for radical resection of PHCC, with right- or left-sided hepatectomy selected depending on the dominant side of cancer progression. If the FLR and liver function are sufficient, trisectionectomy is considered for the complete resection of the extended peripheral spread of PHCC. All hepatectomies were performed after the total bilirubin concentration decreases to <2 mg/dl. When the ratio of the FLR volume to the total liver volume (FLRV/TLV) is <35–40% (indocyanine green retention rate at 15 min (ICGR15) < 10%: FLR/TLV < 35%; ICGR15 10–20%: FLRV/TLV < 40–50%), PVE is performed before radical hepatectomy to reduce the risk of postoperative liver insufficiency [12, 13, 16]. PVE involves performing percutaneous transhepatic portal embolization (PTPE) or transileocolic portal embolization (TIPE) and waiting for a sufficient volume before surgery. TIPE was mainly performed until 2002; however, PTPE was performed after 2002, and TIPE is performed when puncture is difficult.

### Surgery

The liver was transected using the clamp-crushing method, with or without an ultrasonic dissector, after routine application of the intermittent inflow occlusion technique [17]. Concomitant pancreatoduodenectomy (PD) was indicated for the following types of spread in patients with cholangiocarcinoma: (1) widespread cholangiocarcinoma infiltrating the entire extrahepatic bile duct; (2) perihilar cholangiocarcinoma with caudad superficial spread or lymph node (LN) metastasis invading the pancreatic head or duodenum; or (3) distal cholangiocarcinoma with cephalad superficial spread, for which R0 resection was expected [15]. Hepaticojejunostomy was performed using the Roux-en-Y technique. For hepatopancreatoduodenectomy, bilioenteric reconstruction was performed according to the modified Child method. Before closing the abdominal wound, a closed silicone drain was inserted along the cut surface of the liver and behind the bilioenteric anastomosis site.

### Postoperative management

For surveillance, fluid sampled postoperatively from the abdominal drain(s) was submitted for measurement of the total bilirubin level, and bacterial cultures were routinely initiated on days 1, 3, 5, and 7. Blood cultures were performed when a patient developed a fever of >38.0 °C at any time, irrespective of the presence or absence of other infectious sources. The abdominal drainage tubes were removed on postoperative days 5–7 when the drainage fluid was not bilious and bacterial cultures were negative.

### Definitions

Major hepatectomy was defined as liver resection of three or more Couinaud segments, excluding segment 1. The caudate lobe was completely removed en bloc. Mortality included intraoperative death, death within 90 days of surgery, and in-hospital death. Morbidity was defined as a complication occurring within 30 days of surgery or hospitalization. A major complication was defined as a grade III or IV event according to the Clavien–Dindo classification [18]. PHLF, post-hepatectomy bile leakage (PHBL), and postoperative pancreatic fistula (POPF) were diagnosed and graded according to the criteria of the International Study Group of Liver Surgery [19, 20] and the updated criteria of the International Study Group on Pancreatic Surgery [21]. Infectious complications included surgical site infection (SSI), cholangitis, septicemia, and pulmonary and urinary tract infections. SSI was defined according to criteria established by the United States Centers for Disease Control and Prevention [22]. Cholangitis was defined as fever with or without chills; elevated leukocyte counts; increased levels of alkaline phosphatase, γ-glutamyltransferase, or bilirubin; and the absence of urinary tract infection, pneumonia, or SSI. Pneumonia was defined as positive chest radiograph accompanied by leukocytosis.

### Statistical analysis

Continuous and categorical variables were compared using the Mann–Whitney and χ^2^ tests between patients who underwent PVE (PVE [+] group) and those who did not (PVE [−] group). To identify independent risk factors for grade B/C PHLF, multivariable analyses were performed along with a logistic regression analysis with covariates with a cutoff *p* value of 0.10, after eliminating possible confounders. The results are expressed as odds ratios (ORs) with 95% confidence intervals (CIs). Statistical analyses were performed using JMP (ver. 13.2; SAS Institute, Cary, NC, USA) and SPSS Statistics (ver. 25.0; IBM Corp., Armonk, NY, USA). P values of < 0.05 were considered to indicate statistical significance.

## Results

Patient characteristics are summarized in Table 1 according to PBD or non-PBD, with or without the application of PVE. Among the entire cohort (*n* = 240), 186 (76%) received PBD; 121 (65.1%) received ENBD, 34 (18.3%) received PTBD, 9 (4.8%) received EBS, and 22 (11.8%) received a *combination of these*. Among the 186 patients who underwent PBD, 143 (76.9%) had a positive preoperative bile culture and 77 (41.4%) had two or more bacterial species. The median duration of preoperative biliary drainage in patients with PBD was 42 days (range: 4–147 days). The remaining 54 patients did not undergo PBD (non-PBD).

**Table 1:** Patient characteristics

Parameter	PVE (+)			PVE (−)		
	PBD (*n* = 95)	Non-PBD (*n* = 16)	*p*	PBD (*n* = 91)	Non-PBD (*n* = 38)	*p*
Age (years)*	69 (42–83)	72 (53–77)	0.139	71 (41–84)	69 (39–88)	0.368
Sex			0.391			0.225
Male	69 (72.6)	10 (62.5)		65 (71.4)	23 (60.5)	
Female	26 (27.4)	6 (37.5)		26 (28.6)	15 (39.5)	
BMI (kg/m^2^)^*^	21.3 (16.0–31.7)	23.3 (18.6–30.0)	0.045	21.6 (16.7–30.9)	22.8 (15.0–28.4)	0.367
Comorbidity						
Hypertension	30 (31.6)	6 (37.5)	0.774	35 (38.5)	19 (50.0)	0.226
Diabetes mellitus	14 (14.7)	2 (12.5)	1.000	11 (12.1)	2 (5.3)	0.343
Cardiovascular	10 (10.5)	0 (0)	0.352	10 (11.0)	4 (10.5)	1.000
ASA score			0.804			0.861
1	36 (37.9)	5 (31.3)		28 (30.8)	12 (31.6)	
2	51 (53.7)	10 (62.5)		58 (63.7)	23 (60.5)	
3	8 (8.4)	1 (6.3)		5 (5.5)	3 (7.9)	
Total bilirubin (mg/dl)*						
Before PBD	4.9 (0.4–32.0)	–	NA	4.5 (0.4–38.1)	–	NA
Preoperative	0.9 (0.1–2.8)	0.7 (0.3–1.3)	0.074	1.0 (0.4–3.4)	0.8 (0.2–1.6)	0.003
Albumin (g/dl)*	3.6 (2.7–4.6)	3.8 (3.3–4.4)	0.077	3.7 (1.2–4.6)	3.9 (2.9–4.6)	0.002
NLR*	2.20 (0.57–6.85)	2.64 (1.48–6.88)	0.026	2.22 (0.87–10.67)	1.90 (0.77–5.90)	0.101
CONUT score			0.015			0.084
Normal	16 (18.0)	7 (43.8)		32 (40.0)	21 (56.8)	
Light	49 (55.0)	9 (56.2)		42 (47.3)	13 (35.1)	
Moderate	24 (27.0)	0 (0)		14 (15.7)	3 (8.1)	
Severe	0 (0)	0 (0)		0 (0)	0 (0)	
Method of PVE			0.792			NA
PTPE	49 (51.6)	9 (56.3)		–	–	
TIPE	46 (48.4)	7 (43.7)		–	-	
Method of PBD			NA			NA
ENBD	60 (63.2)	–		61 (67.0)	–	
PTBD	22 (23.2)	–		12 (13.2)	–	
EBS	5 (5.3)	–		4 (4.4)	–	
Combined	8 (8.4)	–		14 (15.4)	–	
Duration of PBD (days)*	45 (7–147)	–	NA	39 (4–107)	–	NA
Preoperative bile culture						
Positive	70 (73.7)	–	NA	73 (80.2)	–	NA
Multiple microorganisms	44 (53.7)	–	NA	33 (40.0)	–	NA
Preoperative cholangitis	33 (34.7)	2 (12.5)	0.089	28 (30.8)	1 (2.6)	<0.001
ICGR15 (%)*	11 (3–29)	13 (4–23)	0.501	11 (2–24)	11 (3–18)	0.297
FLRV/TLV (%)*	44 (29–64)	44 (35–50)	0.884	61 (35–87)	65 (36–91)	0.596
FLRV/TLV			0.227			0.722
<40%	28 (29.4)	2 (12.5)		6 (6.7)	3 (7.9)	
≥40%	67 (70.6)	14 (87.5)	0.227	85 (93.3)	35 (92.1)	0.722
Hypertrophy rate** (%)*	8.5 (−1.3–22.8)	8.2 (−2.0–15.4)	0.817	–	–	NA
ICGK-F*	0.067 (0.033–0.214)	0.069 (0.058–0.096)	0.476	0.093 (0.044–0.171)	0.094 (0.045–0.172)	0.263

Values in parentheses represent percentages, unless indicated otherwise

*PVE* portal vein embolization, *PBD* preoperative biliary drainage, *BMI* body mass index, *ASA* American Society of Anesthesiologists, *NA* not applicable, *NLR* neutrophil-to-lymphocyte ratio, *CONUT* controlling nutritional status, *ENBD *endoscopic nasobiliary drainage, *PTBD* percutaneous transhepatic biliary drainage, *EBS* endoscopic biliary stenting, *ICGR15* indocyanine green retention rate at 15 min, *FLRV/TLV* ratio of future liver remnant volume to total liver volume, *ICGK-F* plasma clearance rate of indocyanine green clearance of future liver remnant

*Values represent the median (range)

**FLRV/TLV (%) after PVE-FLRV/TLV (%) before PVE

Among the 111 patients who underwent PVE, there was no significant difference in age, sex, preoperative comorbidities, American Society of Anesthesiologists (ASA) score, preoperative total bilirubin level, or PVE method between those with and without PBD. In contrast, the body mass index (BMI) was significantly higher in the non-PBD group than in the PBD group (23.3 vs. 21.3 kg/m^2^, *p* = 0.045). No difference was observed between the two groups in the preoperative nutritional status of albumin levels. However, the PBD group had significantly more malnourished patients based on the controlling nutritional status (CONUT) score (*p* = 0.015). Additionally, the neutrophil-to-lymphocyte ratio (NLR) was lower in the PBD group (2.20 vs. 2.64, *p* = 0.026) [23]. Preoperative cholangitis was more frequent in patients who underwent PBD than in those who did not (34.7 vs. 12.5%, *p* = 0.089). The ratio of FLR volume to total liver volume (FLRV/TLV), FLRV/TLV < 40%, indocyanine green retention rate at 15 min (ICGR15), and plasma clearance rate of indocyanine green in FLR (ICGK-F) were comparable between the two groups.

Among 129 patients who did not undergo PVE, the PBD group had significantly higher T-bilirubin values and lower albumin values than the non-PBD group. There were no significant differences in other background characteristics between the PBD and non-PBD groups. Preoperative cholangitis occurred more frequently in the PBD group than in the non-PBD group (30.8 vs. 2.6%, *p* < 0.001). The ICGR15, FLRV/TLV, and ICGK-F values were comparable between the two groups.

### Surveillance cultures of preoperative bile and postoperative abdominal drainage fluids

The bacterial species isolated during the final preoperative surveillance of bile cultures are shown in Table 2. Among 186 patients who underwent PBD, the most frequently isolated species were of the following genera: *Enterococcus* (31.7%), *Klebsiella* (21.0%), *Stenotrophomonas* (11.8%), and *Enterobacter* (9.1%). Patients who received ENBD had a higher prevalence of *Enterococcus* species than those who received PTBD in the PVE (+) (33.3 vs. 13.6%, respectively; *p* = 0.099) and PVE (−) groups (36.1 vs. 8.3%, respectively; *p* = 0.089). In contrast, the prevalence of other bacterial species was comparable between ENBD and PTBD groups, with or without PVE.

**Table 2:** Micro-organisms isolated from preoperative bile culture in 186 patients who underwent biliary drainage

	PVE (+)			PVE (−)		
	ENBD (*n* = 60)	PTBD (*n* = 22)	p*	ENBD (*n* = 61)	PTBD (*n* = 12)	p*
Gram-positive bacteria						
* Enterococcus* species	20 (33.3)	3 (13.6)	0.099	22 (36.1)	1 (8.3)	0.089
* Staphylococcus* species	4 (6.7)	3 (13.6)	0.378	(3.3)	1 (8.3)	0.421
* Streptococcus* species	4 (6.7)	0 (0)	0.570	(1.6)	0 (0)	1.000
* Clostridium* species	2 (3.3)	0 (0)	1.000	(3.3)	1 (8.3)	0.421
MRS species	1 (1.7)	1 (4.6)	0.467	(3.3)	0 (0)	1.000
* Bacillus* species	1 (1.7)	0 (0)	1.000	(4.9)	0 (0)	1.000
* Corynebacterium* species	0 (0)	0 (0)	NA	(3.3)	1 (8.3)	1.000
Gram-negative bacteria						
* Klebsiella* species	14 (23.3)	3 (13.6)	0.539	11 (18.0)	1 (8.3)	0.676
* Stenotrophomonas* species	7 (11.7)	2 (9.1)	1.000	5 (8.2)	2 (16.7)	0.323
* Pseudomonas* species	7 (11.7)	0 (0)	0.181	3 (4.9)	2 (16.7)	0.187
* Enterobacter* species	5 (8.3)	1 (4.6)	1.000	6 (9.8)	1 (8.3)	1.000
* Acinetobacter* species	3 (5.0)	4 (18.2)	0.079	4 (6.6)	0 (0)	1.000
* Citrobacter* species	4 (6.7)	0 (0)	0.570	2 (3.3)	0 (0)	1.000
* Morganella* species	3 (5.0)	0 (0)	0.560	1 (1.6)	2 (16.7)	0.068
* Aeromonas* species	2 (3.3)	1 (4.6)	1.000	2 (3.3)	1 (8.3)	0.421
* Escherichia* species	2 (3.3)	0 (0)	1.000	4 (6.6)	0 (0)	1.000
* Serratia* species	2 (0)	0 (0)	1.000	0 (0)	0 (0)	NA
* Fravobacterium* species	1 (1.7)	1 (4.6)	0.467	1 (1.6)	0 (0)	1.000

Values in parentheses represent percentages

*PVE* portal vein embolization, *ENBD* endoscopic nasobiliary drainage, *PTBD* percutaneous transhepatic biliary drainage, *MRS* methicillin-resistant *Staphylococcus*

*Comparison between ENBD and PTBD

The bacterial species isolated from postoperative surveillance cultures of the abdominal drainage fluid within 7 days after surgery are summarized in Table 3. Among the 111 patients who underwent PVE, the frequency of positive cultures was comparable between those with and without PBD (47.3 vs. 43.8%, respectively; *p* = 0.778). Among the 95 patients who underwent PBD, the same bacterial species detected in preoperative bile cultures were isolated from the postoperative abdominal drainage fluid in 15 patients (15.8%). The prevalence of each bacterial species isolated from surveillance cultures of drainage fluids was similar between the PBD and non-PBD groups.

**Table 3:** Micro-organisms isolated from surveillance culture of postoperative abdominal drainage fluid within 7 days after surgery

	PVE (+)			PVE (−)		
	PBD (*n* = 95)	Non-PBD (*n* = 16)	*p*	PBD (*n* = 91)	Non-PBD (*n* = 38)	*p*
Surveillance culture positive	45 (47.3)	7 (43.8)	0.788	36 (39.6)	10 (26.3)	0.152
Same bacterium as preoperative bile culture	15 (15.8)	–	NA	11 (12.1)	–	NA
Gram-positive bacteria						
* Enterococcus* species	16 (16.8)	2 (12.5)	0.663	8 (8.8)	1 (2.6)	0.280
* Staphylococcus* species	3 (3.2)	0 (0)	1.000	2 (2.2)	1 (2.6)	1.000
* Streptococcus* species	3 (3.2)	1 (6.3)	0.468	2 (2.2)	0 (0)	1.000
* Clostridium* species	0 (0)	0 (0)	NA	0 (0)	0 (0)	NA
MRS species	2 (2.1)	1 (6.3)	0.376	8 (8.8)	0 (0)	0.104
* Bacillus* species	2 (2.1)	1 (6.3)	0.376	1 (1.1)	1 (2.6)	0.504
* Corynebacterium* species	0 (0)	0 (0)	NA	1 (1.1)	0 (0)	1.000
Gram-negative bacteria						
* Klebsiella* species	4 (4.2)	0 (0)	0.403	2 (2.2)	0 (0)	1.000
* Stenotrophomonas* species	0 (11.7)	0 (0)	NA	1 (1.1)	0 (0)	1.000
* Pseudomonas* species	6 (6.3)	3 (18.8)	0.120	1 (1.1)	1 (2.6)	0.504
* Enterobacter* species	8 (8.4)	0 (0)	0.560	4 (4.4)	0 (0)	0.319
* Acinetobacter* species	1 (1.1)	0 (0)	1.000	0 (0)	0 (0)	NA
* Citrobacter* species	4 (4.2)	0 (0)	1.000	0 (0)	0 (0)	NA
* Morganella* species	0 (0)	0 (0)	NA	0 (0)	0 (0)	NA
* Aeromonas* species	1 (1.1)	0 (0)	1.000	0 (0)	0 (0)	NA
* Escherichia* species	1 (1.1)	0 (0)	1.000	1 (1.1)	0 (0)	1.000
* Serratia* species	4 (4.2)	0 (0)	1.000	0 (0)	1 (2.6)	0.295
* Fravobacterium* species	0 (0)	0 (0)	NA	0 (0)	0 (0)	NA

Values in parentheses represent percentages

*PVE* portal vein embolization, *PBD* preoperative biliary drainage, *MRS* methicillin-resistant *Staphylococcus*

Among the 129 patients who did not undergo PVE, there was no significant difference in the incidence of positive postoperative surveillance cultures of the abdominal drainage fluid between patients with and without PBD (39.6 vs. 26.3%, respectively; *p* = 0.152), and 11 of 91 patients (12.1%) who underwent PBD had concordant bacterial species in both preoperative bile cultures and abdominal drainage fluid.

### Surgical and postoperative short-term outcomes

The patients’ surgical and short-term postoperative outcomes are presented in Table 4. Among patients who underwent PVE, although the distribution of surgical procedures was similar between those with and without PBD, those with PBD had a higher incidence of concomitant pancreatoduodenectomy (22.1 vs. 0%, *p* = 0.039). However, no significant differences were observed in the duration of surgery, blood loss, or inflow occlusion time between the PBD and non-PBD groups.

**Table 4:** Surgical and postoperative short-term outcomes

Parameter	PVE (+)			PVE (−)		
	PBD (*n* = 95)	Non-PBD (*n* = 16)	*p*	PBD (*n* = 91)	Non-PBD (*n* = 38)	*p*
*Surgical outcomes*						
Operative procedure			0.589			0.086
Right trisectionectomy	2 (2.1)	0 (0)		0 (0)	2 (5.3)	
Left trisectionectomy	7 (7.4)	1 (6.3)		6 (6.6)	0 (0)	
Right hemihepatectomy	82 (86.3)	13 (81.3)		31 (34.1)	12 (31.6)	
Left hemihepatectomy	3 (3.2)	1 (6.3)		52 (57.1)	22 (57.9)	
Central bisectionectomy	1 (1.1)	1 (6.3)		2 (2.2)	2 (5.3)	
Concomitant PD	21 (22.1)	0 (0)	0.039	16 (17.6)	2 (5.3)	0.093
Concomitant vascular resection	17 (17.9)	3 (18.6)	1.000	15 (16.5)	5 (13.2)	0.792
PV	13 (13.7)	1 (6.3)		11 (12.1)	3 (7.9)	
HA	1 (1.1)	0 (0)		4 (4.4)	1 (2.6)	
PV and HA	3 (3.2)	0 (0)		0 (0)	1 (2.6)	
IVC	0 (0)	2 (12.5)		0 (0)	0 (0)	
Duration of operation (min)*	750 (471–1330)	782 (562–950)	0.811	735 (445–1297)	646 (434–1018)	<0.001
Blood loss (ml)*	900 (130–3500)	695 (220–2020)	0.231	850 (120–6600)	570 (150–2700)	<0.001
Inflow occlusion time (min)*	52 (0–111)	60 (40–100)	0.166	60 (0–210)	47 (24–139)	0.016
RBC transfusion	15 (15.8)	2 (12.5)	1.000	12 (13.2)	3 (7.9)	0.551
*Short-term outcomes*						
Mortality	1 (1.1)	0 (0)	1.000	2 (2.2)	0 (0)	1.000
Morbidity	76 (80.0)	11 (68.8)	0.332	72 (79.2)	28 (73.7)	0.497
Major complication	40 (42.1)	6 (37.5)	0.790	29 (31.9)	9 (23.7)	0.353
Surgical site infection	39 (41.1)	5 (31.3)	0.585	23 (25.3)	7 (18.4)	0.496
Incisional	21 (22.1)	4 (25.0)	0.755	9 (9.9)	1 (2.6)	0.279
Organ/space	25 (26.3)	1 (6.3)	0.112	17 (18.7)	7 (18.4)	1.000
PHLF	49 (51.6)	3 (18.8)	0.016	19 (20.9)	8 (21.1)	1.000
Grade B/C	45 (47.4)	3 (18.8)	0.054	10 (11.0)	6 (15.8)	0.559
Grade B/C PHBL	6 (6.3)	1 (6.3)	0.992	5 (5.5)	2 (5.3)	1.000
Postoperative hospital stay (days)*	49 (17–318)	44 (18–65)	0.119	38 (11–198)	46 (12–116)	0.820

Values in parentheses represent percentages, unless indicated otherwise

*PVE* portal vein embolization, *PBD* preoperative biliary drainage, *PD* pancreatoduodenectomy, *PV* portal vein, *HA* hepatic artery, *RBC* red-blood cell, *PHLF* post-hepatectomy liver failure, *PHBL* post-hepatectomy bile leakage, *NA* not applicable

*Values represent the median (range)

Mortality and morbidity rates were comparable between patients with and without PBD (mortality: 1.1 vs. 0%, *p* = 1.000; morbidity: 80.0 vs. 68.8%, *p* = 0.332). Major complications occurred in 40 patients (42.1%) in the PBD group and 6 patients (37.5%) in the no-PBD group (*p* = 0.790). The incidence of grade B/C PHLF was higher in the PBD group than in the non-PBD group (47.4 vs. 18.8%, *p* = 0.054). The median duration of postoperative hospitalization was comparable between the two groups.

Among patients without PVE, those who underwent PBD had longer surgical times (735 vs. 646 min, *p* < 0.001), increased blood loss (850 vs. 570 ml, *p* < 0.001), and longer inflow occlusion times (60 vs. 47 min, *p* = 0.016) than patients without PBD. The postoperative short-term outcomes of the two groups were comparable.

### Risk factors for grade B/C PHLF

Table 5 shows the results of univariate and multivariate analyses of the risk factors for grade B/C PHLF in the entire cohort. The multivariate analysis identified the following risk factors for grade B/C PHLF: application of PVE (OR 3.98, 95% CI 1.88–8.43; *p* < 0.001) and occurrence of organ/space SSI (OR 3.48, 95% CI 1.63–7.42; *p* = 0.001). An ICGK-F < 0.075 (OR 2.03, 95% CI 0.98–4.19; *p* = 0.056) was also associated with the development of grade B/C PHLF. Although univariate analysis identified right-sided hepatectomy as a risk factor for grade B/C PHLF (OR 3.54, 95% CI 1.76–7.11; *p* < 0.001), this parameter, which confounded data interpretation for the application of PVE, was not included in the multivariate analysis. PBD, positive preoperative bile culture, preoperative cholangitis, and preoperative nutritional status did not have any significant negative effects on the occurrence of grade B/C PHLF.

**Table 5:** Univariate and multivariate analyses of risk factors for grade B/C post-hepatectomy liver failure in the entire cohort

Variables	*n*	Univariate		Multivariate	
		Odds ratio	*p*	Odds ratio	*p*
Portal vein embolization					
Yes	111	5.38 (2.83–10.25)	<0.001	3.98 (1.88–8.43)	<0.001
No	129	1.00 (reference)		1.00 (reference)	
Organ/space SSI					
Yes	50	3.87 (2.01–7.47)	<0.001	3.48 (1.63–7.42)	0.001
No	190	1.00 (reference)		1.00 (reference)	
ICGK-F					
≤0.075	117	3.78 (2.03–7.04)	<0.001	2.03 (0.98–4.19)	0.056
>0.075	123	1.00 (reference)		1.00 (reference)	
Diabetes					
Yes	29	2.16 (0.97–4.82)	0.060	2.26 (0.91–5.62)	0.080
No	211	1.00 (reference)		1.00 (reference)	
Blood loss					
≥1 L	84	2.38 (1.33–4.29)	0.004	1.45 (0.68–3.07)	0.336
<1 L	156	1.00 (reference)		1.00 (reference)	
Duration of operation					
≥720 min	122	2.29 (1.26–4.15)	0.006	1.39 (0.65–2.95)	0.392
<720 min	118	1.00 (reference)		1.00 (reference)	
Hepatopancreatoduodenectomy					
Yes	39	1.98 (0.94–3.99)	0.072	1.34 (0.31–1.81)	0.515
No	201	1.00 (reference)		1.00 (reference)	
Preoperative biliary drainage					
Yes	186	2.10 (0.96–4.59)	0.063	1.11 (0.45–2.71)	0.821
No	54	1.00 (reference)		1.00 (reference)	
Type of hepatectomy					
Right-sided	143	3.54 (1.76–7.11)	<0.001		
Central	6	3.29 (0.54–20.0)	0.195		
Left-sided	91	1.00 (reference)			
Preoperative bile culture					
Positive	143	0.99 (0.55–1.77)	0.968		
Negative or no drainage	97	1.00 (reference)			
Preoperative cholangitis					
Yes	64	0.99 (0.52–1.90)	0.980		
No	176	1.00 (reference)			
Albumin					
≥3.4 g/dl	194	1.39 (0.64–3.99)	0.402		
<3.4 g/dl	46	1.00 (reference)			
NLR					
≥2.36	102	1.26 (0.71–2.26)	0.431		
<2.36	133	1.00 (reference)			
CONUT					
Moderate and severe	42	1.07 (0.50–2.28)	0.862		
Normal and light	189	1.00 (reference)			

Values in parentheses represent 95% confidence intervals

*SSI* surgical site infection, *ICGK-F* plasma clearance rate of indocyanine green clearance of future liver remnant, *NLR* neutrophil-to-lymphocyte ratio, *CONUT* controlling nutritional status

### Risk factors for grade B/C PHLF in patients who underwent PVE

The results of the univariate and multivariate analyses of the risk factors for grade B/C PHLF in the 111 patients who underwent PVE are shown in Table 6. The multivariate analysis revealed that the occurrence of organ/space SSI was the only independent risk factor for grade B/C PHLF (OR 4.48, 95% CI 1.59–12.66; *p* = 0.005). Consistent with the results of the analysis of the entire cohort, PBD, preoperative bactibilia, preoperative cholangitis, and preoperative nutritional status were not identified as independent risk factors for grade B/C PHLF.

**Table 6:** Univariate and multivariate analyses of risk factors for grade B/C post-hepatectomy liver failure in 111 patients who underwent portal vein embolization

Variables	*n*	Univariable		Multivariable	
		Odds ratio	*p*	Odds ratio	*p*
Organ/space SSI					
Yes	26	5.24 (1.98–13.91)	<0.001	4.48 (1.59–12.66)	0.005
No	85	1.00 (reference)		1.00 (reference)	
Preoperative biliary drainage					
Yes	95	3.90 (1.04–14.58)	0.043	3.30 (0.82–13.33)	0.093
No	16	1.00 (reference)		1.00 (reference)	
Duration of operation					
≥720 min	64	1.94 (0.89–4.21)	0.095	1.49 (0.62–3.56)	0.220
<720 min	47	1.00 (reference)		1.00 (reference)	
Hepatopancreatoduodenectomy					
Yes	21	2.55 (0.96–6.79)	0.060	1.51 (0.48–4.76)	0.481
No	90	1.00 (reference)		1.00 (reference)	
Albumin					
≥3.4 g/dl	87	2.80 (1.02–7.73)	0.047	2.88 (0.94–8.80)	0.063
<3.4 g/dl	24	1.00 (reference)		1.00 (reference)	
Preoperative bile culture					
Positive	70	1.12 (0.51–2.45)	0.772		
Negative or no drainage	41	1.00 (reference)			
Preoperative cholangitis					
Yes	35	0.69 (0.30–1.57)	0.380		
No	76	1.00 (reference)			
ICGK-F					
<0.075	81	1.45 (0.61–3.44)	0.396		
≥0.075	30	1.00 (reference)			
Type of hepatectomy					
Right-sided	97	0.50 (0.15–1.69)	0.266		
Central	2	0.71 (0.04–14.35)	0.826		
Left-sided	12	1.00 (reference)			
NLR					
≥2.36	102	0.98 (0.46–2.12)	0.980		
<2.36	133	1.00 (reference)			
CONUT					
Moderate and severe	24	0.54 (0.21–1.40)	0.203		
Normal and light	81	1.00 (reference)			

Values in parentheses represent 95% confidence intervals

*SSI* surgical site infection, *ICGK-F* plasma clearance rate of indocyanine green clearance of future liver remnant, *NLR* neutrophil-to-lymphocyte ratio, *CONUT* controlling nutritional status

### Surgical and postoperative short-term outcomes associated with hypertrophy rate after PVE and PBD

The characteristics as well as the surgical and postoperative short-term outcomes of the 88 patients who underwent PVE and PBD are shown in Table 7. We excluded seven patients in whom the intended surgical procedure was changed after PVE. Parameters were compared between 43 patients with hypertrophy rates ≥ 25% (FLRV/TLV [%] after PVE/FLRV/TLV [%] before PVE × 100 − 100) [24] and 45 patients with hypertrophy rates < 25%. Although the frequencies of positive preoperative bile culture were comparable between the two groups (data not shown), the frequency of preoperative cholangitis was significantly higher in patients with hypertrophy rates < 25% than in those with hypertrophy rates ≥ 25% (48.9 vs. 20.9%, *p* = 0.006).

**Table 7:** Comparison of characteristics, surgical, and short-term outcomes by hypertrophy rate in 88 patients who underwent both of portal vein embolization and preoperative biliary drainage

Parameter	Hypertrophy rate ≥ 25% (*n* = 43)	Hypertrophy rate < 25% (*n* = 45)	*p*
*Characteristics*			
Method of biliary drainage			0.401
ENBD	29 (67.4)	27 (60.0)	
PTBD	10 (23.3)	11 (24.4)	
EBS	3 (7.0)	2 (4.4)	
Combined	1 (2.3)	5 (11.1)	
Duration of drainage (days)*	45 (13–86)	46 (7–112)	0.101
Preoperative bile culture positive	30 (70.0)	36 (80.0)	0.268
Multiple microorganisms	17 (46.0)	24 (60.0)	0.217
Preoperative cholangitis	9 (20.9)	22 (48.9)	0.006
ICGR15 (%)*	11 (4–27)	10 (3–29)	0.654
FLRV/TLV (%)*	45 (32–60)	42 (29–57)	0.006
ICGK-F*	0.062 (0.034–0.121)	0.066 (0.033–0.214)	0.121
*Surgical outcomes*			
Surgical procedure			0.442
Right trisectionectomy	0 (0)	1 (2.2)	
Left trisectionectomy	2 (4.7)	4 (8.9)	
Right hemihepatectomy	41 (95.4)	40 (88.9)	
Left hemihepatectomy	0 (0)	0 (0)	
Central bisectionectomy	0 (0)	0 (0)	
Concomitant PD	10 (23.3)	10 (22.2)	1.000
Concomitant vascular resection	6 (14.0)	10 (22.2)	0.410
Portal vein	4 (9.3)	8 (17.8)	
Hepatic artery	0 (0)	1 (2.2)	
Portal vein and hepatic artery	2 (4.7)	1 (2.2)	
Duration of operation (min)*	732 (574–1305)	750 (471–1330)	0.649
Blood loss (ml)*	890 (320–2100)	922 (130–3500)	0.825
Inflow occlusion time (min)*	45 (24–109)	56 (0–111)	0.470
RBC transfusion	5 (11.6)	8 (17.8)	0.551
*Short-term outcomes*			
Mortality	0 (0)	1 (2.2)	1.000
Morbidity	36 (83.7)	35 (77.8)	0.592
Major complication	14 (32.6)	22 (48.9)	0.119
Surgical site infection	19 (44.2)	17 (37.8)	0.541
Incisional	11 (25.6)	8 (17.8)	0.442
Organ/space	10 (23.3)	13 (28.9)	0.548
PHLF	21 (48.8)	24 (53.3)	0.673
Grade B/C	21 (48.8)	20 (44.4)	0.680
Grade B/C PHBL	3 (7.0)	2 (4.4)	0.673
Surveillance culture of drainage fluid positive	25 (58.1)	17 (37.8)	0.056
Same bacterium as preoperative bile culture	7 (16.3)	7 (15.6)	1.000
Postoperative hospital stay (days)*	49 (17–130)	48 (21–288)	0.851

Values in parenthesis represent percentage, unless indicated otherwise

*ENBD* endoscopic nasobiliary drainage, *PTBD* percutaneous transhepatic biliary drainage, *EBS* endoscopic biliary stenting, *ICGR15* indocyanine green retention rate at 15 min, *FLRV/TLV* ratio of future liver remnant volume to total liver volume, *ICGK-F* plasma clearance rate of indocyanine green clearance of future liver remnant, *PD* pancreatoduodenectomy, *RBC* red-blood cell, *PHLF* post-hepatectomy liver failure, *PHBL* post-hepatectomy bile leakage

*Values represent the median (range)

The frequency of positive surveillance cultures within 7 days after surgery in patients with hypertrophy rates ≥ 25% was higher than that in patients with hypertrophy rates < 25% (58.1 vs. 37.8%, *p* = 0.056). The mortality and morbidity rates, frequencies of major complications, and grade B/C PHLF were not significantly different between the two groups (Table 7).

## Discussion

This study revealed that PBD, which is frequently associated with positive bile cultures, did not significantly influence the occurrence of postoperative complications, particularly PHLF, which is the most common and critical complication of major hepatectomy for PHCC. Septic complications and severe PHLF are critical and often lead to deteriorating outcomes in patients undergoing major hepatectomy [25, 26]. Therefore, effective treatment and management strategies are required to prevent these complications.

PHCC patients typically develop obstructive jaundice. Therefore, PBD is necessary to alleviate jaundice, restore the liver function, and prevent PHLF. For example, Belghiti et al. [6] found that the mortality rate of patients with obstructive jaundice who underwent hepatectomy was significantly higher than that of patients with a normal liver function. Furthermore, Farges et al. [11] reported that PBD increased the mortality rate in patients who underwent left-sided hepatectomy, whereas patients who underwent right-sided hepatectomy with PBD experienced a lower mortality rate. In this series, liver failure was the most frequent cause of mortality in patients who underwent right-sided hepatectomy and was more frequently experienced by patients who did not undergo PBD than by those who underwent PBD [11]. Subgroup analyses were performed for right- and left-sided hepatectomy. However, PBD was not identified as an independent risk factor for grade B/C PHLF in either group (Supplementary Tables 1 and 2).

These reports indicate that PBD is necessary to effectively manage patients scheduled to undergo major hepatectomy. Moreover, we showed that the mortality rate of 186 patients who underwent major hepatectomy was relatively low (1.6%) when PBD was routinely applied to manage obstructive jaundice. In contrast, PBD or bactibilia was associated with increased postoperative mortality, morbidity, and infectious complications. For example, Ferrero et al. [8] found that although PBD did not reduce mortality or morbidity rates, it did increase the incidence of infectious complications after hepatectomy. Furthermore, PBD is an independent risk factor for infectious complications. Ramanathan et al. [9] found that PBD increased mortality and morbidity rates after hepatectomy for perihilar and intrahepatic cholangiocarcinoma. However, it remains unclear whether preoperative surveillance bile cultures and the selection of AMP were appropriately performed in these studies [8, 9].

In contrast, Sugawara et al. [10] did not find a significant association between PBD and the development of postoperative infectious complications, suggesting the importance of selecting an appropriate AMP based on perioperative surveillance of bile cultures. Takara et al. [27] found that using an appropriate AMP (including vancomycin) tailored to bile culture findings, hepatectomy could be performed with low mortality (0%) and a low incidence of SSI in patients with preoperative biliary MRSA contamination. Consistent with the results of these studies [10, 27], we demonstrate here that PBD was not significantly associated with PHLF through the use of appropriate AMP targeted to bacteria isolated from preoperative surveillance bile cultures.

The methodology required to perform preoperative surveillance of bile cultures may influence postoperative outcomes. For example, Sakata et al. [28] found that a positive preoperative bile culture is an independent risk factor for SSI, and that preoperative cholangitis independently increases the mortality rate after major hepatectomy with concomitant bile duct resection. Although 71 patients (87.7%) who underwent PBD had preoperative bile cultures, the frequency and timing of surveillance were unclear [28]. In contrast, we found that a positive preoperative bile culture or preoperative cholangitis was not significantly associated with the occurrence of grade B/C PHLF.

In 26 of 143 patients (18.2%) with positive preoperative bile cultures, the postoperative surveillance cultures of the abdominal drainage fluid contained the same bacterial species identified in the preoperative bile cultures. Notably, the preoperative bile culture results of patients who developed preoperative cholangitis did not differ significantly from those of the other groups (Supplementary Table 3). Additionally, the same bacterial species were identified in the preoperative bile culture and postoperative surveillance culture in 9 of 64 patients (14.1%). These results suggest that periodic preoperative surveillance cultures of bile are important for the appropriate selection of AMP, as bacteria in the bile may be replaced by other species during PBD, particularly when antibiotics are used to treat preoperative cholangitis.

Here, we focused on the effect of PBD on the occurrence of postoperative complications, particularly PHLF, in patients who underwent PVE before major hepatectomy. These patients were initially judged to have an inadequate FLR. Postoperative outcomes were compared between the patients with higher and lower hypertrophy rates after PVE. Thus, among the risk factors for grade B/C PHLF in 111 patients who underwent PVE, organ/space SSI was the only independent risk factor. Patients with lower hypertrophy rates after PVE (<25%) experienced a higher incidence of preoperative cholangitis and lower FLRV/TLV than those with higher hypertrophy rates (≥25%). Furthermore, the postoperative outcomes, including the incidence of PHLF, were comparable between the two groups, and preoperative cholangitis and lower hypertrophy rates after PVE (<25%) were not independent risk factors for PHLF (Supplementary Table 4). Thus, preoperative cholangitis may inhibit sufficient hypertrophy of the liver parenchyma after PVE. If a certain FRLV is achieved, the influence of a lower hypertrophy rate after PVE on postoperative outcomes is limited. These results suggest that it is more important to obtain a sufficient FLRV at the time of radical hepatectomy than to obtain a higher rate of hypertrophy after PVE. However, regarding the evaluation of FLRV, Ikehara et al. [29] reported that an appropriate method should be selected based on the patient’s body mass. Based on these findings, future studies should consider this factor, particularly in marginal cases.

Among the entire cohort and patients who underwent PVE, organ/space SSI was identified as an independent risk factor for grade B/C PHLF, whereas PBD was not a risk factor in either population. Furthermore, the bacterial species isolated from the postoperative surveillance cultures of the abdominal drainage fluid were concordant with those isolated through preoperative bile cultures in 16% of the patients with PVE and 12% of the patients without PVE. These findings suggest that the appropriate selection and use of AMP, according to the results of preoperative bile culture, reduces postoperative organ/space SSI, which affects the occurrence of PHLF.

Itoyama et al. [30] reported that 41.3% of 121 patients who underwent PD had intraperitoneal contamination on postoperative day 3, and 78.0% of patients had positive intraoperative bile cultures. Furthermore, they found a high concordance (94.9%) between bacterial species isolated from intraoperative bile cultures and those isolated from abdominal drainage fluid. The discrepancy in the similarities between bile and abdominal fluid cultures between the study by Itoyama et al. [30] and the present study may be due to differences in surgical procedures and strategies for selecting AMP. Although the results of intraoperative bile culture may be useful for selecting antibiotics for the treatment of postoperative infectious complications, the importance of preoperative bile culture for selecting AMP is immutable for preventing postoperative organ/space SSIs.

The present study was associated with several limitations. First, the relatively long study period may raise concerns about the influence of changes in perioperative management on postoperative outcomes. Second, this retrospective study analyzed a relatively small patient population treated at a single institution. Third, to examine the impact of PBD, it is necessary to compare patients with biliary obstruction who underwent PBD with those who did not. However, we believe that our results represent a significant contribution to the field because we showed that PBD did not adversely influence postoperative outcomes with appropriate perioperative surveillance and management of patients with PHCC regardless of whether they underwent PVE.

In conclusion, PBD did not negatively influence postoperative short-term outcomes, including PHLF, when we employed AMP targeting drug-sensitive bacteria isolated from bile cultures of patients who underwent major hepatectomy for PHCC. Therefore, to reduce PHLF, it is critically important to prevent postoperative organ/space SSI through appropriate AMP selection according to perioperative surveillance culture results, particularly in patients undergoing PVE with a small FLR.

## Supplementary Information

Below is the link to the electronic supplementary material.

Supplementary file1 (DOC 74 KB)

Supplementary file2 (DOC 73 KB)

Supplementary file3 (DOC 42 KB)

Supplementary file4 (DOC 59 KB)
