# Supplementary material for: Impact of preoperative biliary drainage on postoperative outcomes in patients who undergo major hepatectomy after portal vein embolization for perihilar cholangiocarcinoma
Source: Surg Today. 2025 Jul 8;55(12):1883–95. doi: 10.1007/s00595-025-03080-4 (PMC12602568; doi:10.1007/s00595-025-03080-4)
Supplement: Supplementary file 1 — Supplementary file1 (DOC 74 KB) [file 595_2025_3080_MOESM1_ESM.doc]

| **Supplementary Table 1.** Univariate and multivariate analyses of risk factors for grade B/C post-hepatectomy liver failure in 143 patients who underwent right-sided hepatectomy | | | | | |  |
| --- | --- | --- | --- | --- | --- | --- |
| **Variables** | **n** | **Univariable** | |  | **Multivariable** | |
|  |  | **Odds ratio** | ***P*** |  | **Odds ratio** | ***P*** |
| Portal vein embolization |  |  |  |  |  |  |
| Yes | 97 | 2.53 (1.12-5.68) | 0.025 |  | 1.98 (0.80-4.95) | 0.14 |
| No | 46 | 1.00 (reference) |  |  | 1.00 (reference) |  |
| Organ/space SSI |  |  |  |  |  |  |
| Yes | 29 | 7.69 (3.08-19.23) | < 0.001 |  | 6.67 (2.54-17.54) | < 0.001 |
| No | 114 | 1.00 (reference) |  |  | 1.00 (reference) |  |
| Duration of operation |  |  |  |  |  |  |
| ≥ 720 min | 71 | 2.16 (1.07-4.37) | 0.032 |  | 1.33 (0.56-3.16) | 0.525 |
| < 720 min | 72 | 1.00 (reference) |  |  | 1.00 (reference) |  |
| Hepatopancreatoduodenectomy |  |  |  |  |  |  |
| Yes | 24 | 2.62 (1.07-6.41) | 0.034 |  | 1.55 (0.54-4.46) | 0.421 |
| No | 119 | 1.00 (reference) |  |  | 1.00 (reference) |  |
| ICGK-F |  |  |  |  |  |  |
| < 0.075 | 104 | 2.17 (0.93-5.03) | 0.072 |  | 1.60 (0.63-4.07) | 0.32 |
| ≥ 0.075 | 39 | 1.00 (reference) |  |  | 1.00 (reference) |  |
| Blood loss |  |  |  |  |  |  |
| ≥ 1L | 49 | 1.92 (0.94-3.92) | 0.074 |  | 1.18 (0.48-2.88) | 0.724 |
| < 1L | 94 | 1.00 (reference) |  |  | 1.00 (reference) |  |
| Preoperative biliary drainage |  |  |  |  |  |  |
| Yes | 115 | 2.27 (0.85-6.02) | 0.1 |  | 1.52 (0.50-4.63) | 0.458 |
| No | 28 | 1.00 (reference) |  |  | 1.00 (reference) |  |
| Diabetes |  |  |  |  |  |  |
| Yes | 21 | 1.86 (0.73-4.76) | 0.192 |  |  |  |
| No | 122 | 1.00 (reference) |  |  |  |  |
| Preoperative bile culture |  |  |  |  |  |  |
| Positive | 90 | 1.07 (0.53-2.19) | 0.847 |  |  |  |
| Negative or no drainage | 53 | 1.00 (reference) |  |  |  |  |
| Preoperative cholangitis |  |  |  |  |  |  |
| Yes | 38 | 0.96 (0.44-2.09) | 0.909 |  |  |  |
| No | 105 | 1.00 (reference) |  |  |  |  |

Values in parentheses represent 95% confidence intervals.

*SSI* surgical site infection, *ICGK-F* plasma clearance rate of indocyanine green clearance of future liver remnant.
