# Supplementary material for: Impact of preoperative biliary drainage on postoperative outcomes in patients who undergo major hepatectomy after portal vein embolization for perihilar cholangiocarcinoma
Source: Surg Today. 2025 Jul 8;55(12):1883–95. doi: 10.1007/s00595-025-03080-4 (PMC12602568; doi:10.1007/s00595-025-03080-4)
Supplement: Supplementary file 2 — Supplementary file2 (DOC 73 KB) [file 595_2025_3080_MOESM2_ESM.doc]

| **Supplementary Table 2.** Univariate and multivariate analyses of risk factors for grade B/C post-hepatectomy liver failure in 91 patients who underwent left-sided hepatectomy | | | | | | |
| --- | --- | --- | --- | --- | --- | --- |
| **Variables** | **n** | **Univariable** | |  | **Multivariable** | |
|  |  | **Odds ratio** | ***P*** |  | **Odds ratio** | ***P*** |
| Portal vein embolization |  |  |  |  |  |  |
| Yes | 12 | 20.8 (4.81-90.91) | < 0.001 |  | 17.86 (3.12-100.00) | 0.001 |
| No | 79 | 1.00 (reference) |  |  | 1.00 (reference) |  |
| Duration of operation |  |  |  |  |  |  |
| ≥ 720 min | 46 | 3.40 (0.86-13.51) | 0.082 |  | 1.34 (0.20-9.01) | 0.764 |
| < 720 min | 45 | 1.00 (reference) |  |  | 1.00 (reference) |  |
| ICGK-F |  |  |  |  |  |  |
| < 0.075 | 104 | 7.35 (1.84-29.36) | 0.005 |  | 3.04 (0.43-21.71) | 0.268 |
| ≥ 0.075 | 39 | 1.00 (reference) |  |  | 1.00 (reference) |  |
| Blood loss |  |  |  |  |  |  |
| ≥ 1L | 30 | 5.18 (1.42-18.87) | 0.013 |  | 4.72 (0.63-35.71) | 0.13 |
| < 1L | 61 | 1.00 (reference) |  |  | 1.00 (reference) |  |
| Preoperative biliary drainage |  |  |  |  |  |  |
| Yes | 68 | 1.02 (0.25-4.13) | 0.981 |  | 0.35 (0.05-2.54) | 0.301 |
| No | 23 | 1.00 (reference) |  |  | 1.00 (reference) |  |
| Hepatopancreatoduodenectomy |  |  |  |  |  |  |
| Yes | 12 | 0.56 (0.07-4.78) | 0.598 |  |  |  |
| No | 79 | 1.00 (reference) |  |  |  |  |
| Organ/space SSI |  |  |  |  |  |  |
| Yes | 19 | 1.31 (0.32-5.41) | 0.707 |  |  |  |
| No | 72 | 1.00 (reference) |  |  |  |  |
| Diabetes |  |  |  |  |  |  |
| Yes | 8 | 2.43 (0.43-13.70) | 0.314 |  |  |  |
| No | 83 | 1.00 (reference) |  |  |  |  |
| Preoperative bile culture |  |  |  |  |  |  |
| Positive | 51 | 0.51 (0.15-1.76) | 0.287 |  |  |  |
| Negative or no drainage | 40 | 1.00 (reference) |  |  |  |  |
| Preoperative cholangitis |  |  |  |  |  |  |
| Yes | 25 | 1.38 (0.38-5.08) | 0.626 |  |  |  |
| No | 66 | 1.00 (reference) |  |  |  |  |

Values in parentheses represent 95% confidence intervals.

*ICGK-F* plasma clearance rate of indocyanine green clearance of future liver remnant, *SSI* surgical site infection.
