# Supplementary material for: Impact of preoperative biliary drainage on postoperative outcomes in patients who undergo major hepatectomy after portal vein embolization for perihilar cholangiocarcinoma
Source: Surg Today. 2025 Jul 8;55(12):1883–95. doi: 10.1007/s00595-025-03080-4 (PMC12602568; doi:10.1007/s00595-025-03080-4)
Supplement: Supplementary file 3 — Supplementary file3 (DOC 42 KB) [file 595_2025_3080_MOESM3_ESM.doc]

| **Supplementary Table 3.** Micro-organisms isolated from preoperative bile culture in 64 patients with preoperative cholangitis | | |
| --- | --- | --- |
|  | **Preoperative cholangitis (+)** | |
|  | **(n = 64)** | |
| Gram-positive bacteria | | |
| *Enterococcus* species | 28 | (43.8) |
| *Staphylococcus* species | 4 | (6.3) |
| *Streptococcus* species | 1 | (1.6) |
| *Clostridium* species | 1 | (1.6) |
| MRS species | 4 | (6.3) |
| *Bacillus* species | 1 | (1.6) |
| *Corynebacterium* species | 0 | (0) |
| Gram-negative bacteria | | |
| *Klebsiella* species | 16 | (25.0) |
| *Stenotrophomonas* species | 10 | (15.6) |
| *Pseudomonas* species | 2 | (3.1) |
| *Enterobacter* species | 9 | (14.1) |
| *Acinetobacter* species | 6 | (9.4) |
| *Citrobacter* species | 4 | (6.3) |
| *Morganella* species | 3 | (4.7) |
| *Aeromonas* species | 5 | (7.8) |
| *Escherichia* species | 5 | (7.8) |
| *Serratia* species | 0 | (0) |
| *Fravobacterium* species | 1 | (1.6) |

Values in parentheses represent percentages.

*MRS* Methicillin-resistant *Staphylococcus.*
