# Supplementary material for: Impact of preoperative biliary drainage on postoperative outcomes in patients who undergo major hepatectomy after portal vein embolization for perihilar cholangiocarcinoma
Source: Surg Today. 2025 Jul 8;55(12):1883–95. doi: 10.1007/s00595-025-03080-4 (PMC12602568; doi:10.1007/s00595-025-03080-4)
Supplement: Supplementary file 4 — Supplementary file4 (DOC 59 KB) [file 595_2025_3080_MOESM4_ESM.doc]

| **Supplementary Table 4.** Univariate and multivariate analyses of risk factors for grade B/C post-hepatectomy liver failure in the entire cohort | | | | | | |
| --- | --- | --- | --- | --- | --- | --- |
| **Variables** | **n** | **Univariate** | |  | **Multivariate** | |
|  |  | **Odds ratio** | ***P*** |  | **Odds ratio** | ***P*** |
| Portal vein embolization  Yes  No | 111  129 | 5.38 (2.83-10.25)  1.00 (reference) | < 0.001 |  | 3.92 (1.81-8.47)  1.00 (reference) | 0.001 |
| Organ/space SSI  Yes  No | 50  190 | 3.87 (2.01-7.47)  1.00 (reference) | < 0.001 |  | 3.46 (1.62-7.41)  1.00 (reference) | 0.001 |
| ICGK-F  ≤ .075  > .075 | 117  123 | 3.78 (2.03-7.04)  1.00 (reference) | < 0.001 |  | 2.00 (0.97-4.14)  1.00 (reference) | 0.061 |
| Diabetes  Yes  No | 29  211 | 2.16 (0.97-4.82)  1.00 (reference) | 0.060 |  | 2.29 (0.92-5.68)  1.00 (reference) | 0.075 |
| Blood loss  ≥ 1L  < 1L | 84  156 | 2.38 (1.33-4.29)  1.00 (reference) | 0.004 |  | 1.45 (0.68-3.10)  1.00 (reference) | 0.335 |
| Duration of operation  ≥ 720 min  < 720 min | 122  118 | 2.29 (1.26-4.15)  1.00 (reference) | 0.006 |  | 1.36 (0.64-2.90)  1.00 (reference) | 0.421 |
| Hepatopancreatoduodenectomy  Yes  No | 39  201 | 1.98 (0.94-3.99)  1.00 (reference) | 0.072 |  | 1.50 (0.61-3.70)  1.00 (reference) | 0.376 |
| Preoperative biliary drainage  Yes  No | 186  54 | 2.10 (0.96-4.59)  1.00 (reference) | 0.063 |  | 1.06 (0.43-2.64)  1.00 (reference) | 0.892 |
| Preoperative cholangitis and lower hypertrophy rates after PVE (<25%)  Yes  No | 64  176 | 3.11 (1.28-7.59)  1.00 (reference) | 0.013 |  | 1.17 (0.41-3.37)  1.00 (reference) | 0.766 |
| Type of hepatectomy  Right-sided  Central  Left-sided | 143  6  91 | 3.54 (1.76-7.11)  3.29 (0.54-20.0)  1.00 (reference) | < 0.001  0.195 |  |  |  |

| **Supplementary Table 4** continued | | | | | | |
| --- | --- | --- | --- | --- | --- | --- |
| **Variables** | ***n*** | **Univariate** | |  | **Multivariate** | |
|  |  | **Odds ratio** | ***P*** |  | **Odds ratio** | ***P*** |
| Preoperative bile culture  Positive  Negative or no drainage | 143  97 | 0.99 (0.55-1.77)  1.00 (reference) | 0.968 |  |  |  |
| Albumin  ≥ 3.4 g/dl  < 3.4 g/dl | 194  46 | 1.39 (0.64-3.99)  1.00 (reference) | 0.402 |  |  |  |
| NLR  ≥ 2.36  < 2.36 | 102  133 | 1.26 (0.71-2.26)  1.00 (reference) | 0.431 |  |  |  |
| CONUT  Moderate and severe  Normal and light | 42  189 | 1.07 (0.50-2.28)  1.00 (reference) | 0.862 |  |  |  |

Values in parentheses represent 95% confidence intervals.

*SSI* surgical site infection, *ICGK-F* plasma clearance rate of indocyanine green clearance of future liver remnant, *NLR* neutrophil-to-lymphocyte ratio, *CONUT* controlling nutritional status.
